# 3D Shape Measurement of Aeroengine Blade Based on Fringe Projection Profilometer Improved by Multi-Layer Concentric Ring Calibration

**DOI:** 10.3390/s24092810

**Published:** 2024-04-28

**Authors:** Ze Chen, Yuhang Ju, Chuanzhi Sun, Yinchu Wang, Yongmeng Liu, Jiubin Tan

**Affiliations:** 1 Center of Ultra-Precision Optoelectronic Instrument Engineering, Harbin Institute of Technology, Harbin 150080, China; 22b901036@stu.hit.edu.cn (Z.C.); jyh2125363958@163.com (Y.J.); jbtan@hit.edu.cn (J.T.); 2 Key Lab of Ultra-Precision Intelligent Instrumentation Engineering, Harbin Institute of Technology, Ministry of Industry and Information Technology, Harbin 150080, China; 3 School of Automation, Southeast University, Nanjing 210096, China; martinwang52@163.com

**Keywords:** multi-layer concentric ring calibration, fringe projection, aeroengine blades

## Abstract

The precision requirements for aeroengine blade machining are exceedingly stringent. This study aims to improve the accuracy of existing aeroengine blade measurement methods while achieving comprehensive measurement. Therefore, this study proposes a new concentric ring calibration method and designs a multi-layer concentric ring calibration plate. The effectiveness of this calibration method was verified through actual testing of standard ball gauges. Compared with the checkerboard-grid calibration method, the average deviation of the multilayer concentric ring calibration method for measuring the center distance of the standard sphere is 0.02352, which improves the measurement accuracy by 3–4 times. On the basis of multi-layer concentric ring calibration, this study builds a fringe projection profiler based on the three-frequency twelve-step phase shift method. Compared with the CMM, the average deviation of the blade chord length measured by this solution is 0.064, which meets the measurement index requirements of aeroengine fan blades.

## 1. Introduction

Blades are key parts of aeroengines, with complex structures and high processing-accuracy requirements. The manufacturing quality of the blades has a direct impact on the performance of the aeroengine. Consequently, it is imperative to employ precise and efficient measurement methods to assess whether the various indicators of the blades meet the stipulated requirements. This ensures that the optimal performance of the aeroengine is maintained [1].

Traditional contact measurement systems, such as three-dimensional coordinate measuring machines, are used to measure aircraft-engine blades. The three-coordinate measurement method [2,3] is currently one of the methods with the highest accuracy in blade measurement. However, this method suffers from low measurement efficiency, inadequate adaptability to varying environments, and the potential risk of surface damage. With the development of optoelectronic technology, non-contact optical measurement technology has developed rapidly. It mainly includes holographic interferometry [4], binocular stereo vision [5], a LiDAR–camera system [6], laser triangulation [7], and structured light [8,9]. Among them, the fringe projection profilometer [10] is a non-contact measurement method that can take into account both measurement accuracy and measurement efficiency. The entire measurement system includes cameras, lenses, and projectors. The total measurement system costs about USD 4000, and the construction cost is significantly lower than other measurement solutions, such as three-dimensional coordinate measuring machines. This method obtains the spatial point cloud information of the leaves through camera calibration, phase extraction, stereo matching, and depth calculation.

However, the actual measurement results of the fringe projection profilometer are limited by the camera calibration accuracy and phase extraction accuracy. Although the traditional checkerboard calibration method [11] is robust, the grayscale transition between black and white squares on the calibration board is not smooth. This defect will lead to inaccurate image binarization and increased errors in corner point detection of the calibration plate. Coded concentric rings [12] are a camera calibration method proposed in recent years. It achieves the positioning of calibration feature points through the radial line fitting center method, which can well reduce the projection centrifugal error. However, this method uses the Hough transform to identify straight line segments, which results in a long calibration process and high computational complexity. At the same time, this method determines the coordinates of the calibration feature points by fitting the connecting lines of intersection points in the Hough parameter space. The parameter space may be affected by spurious peaks caused by noise, which may lead to the introduction of incorrect intersection data. In response to the above problems, we introduced the ring feature to avoid the impact of uneven grayscale transition on camera calibration. We reduce the projection centrifugal error, reduce the computational complexity, and improve the calibration speed by introducing multi-layer concentric rings.

The effectiveness of multi-layer concentric ring calibration mainly depends on the accuracy of extracting the center of the ellipse. Existing commonly used ellipse detection methods can be simply divided into two categories: (1) Hough transform and (2) edge following. McLaughlin et al. [13] improved the standard HT [14] to improve the detection performance and became the benchmark for ellipse detection methods. However, this method is still not efficient enough and often produces false detections. Patraucean et al. proposed to use improved LSD to detect LS and then iteratively search the remaining LS from the starting point and end point of the detected LS. Although this method achieves good detection results, it ignores the overall situation, which may easily produce false positives [15]. C. Lu [16] proposed a new ellipse detection algorithm, based on Patraucean et al., by separating the formation of arc-support line segments from grouping. This method is efficient, accurate, and has good robustness. However, since this method is oriented to ellipse detection in complex environments, the computational complexity is high. And in actual calibration scenarios, the multi-layer concentric rings on the calibration plate have low background interference. Therefore, this study tailors the ellipse detection algorithm and proposes a new multi-layer concentric ring calibration method by designing K-means circle-center approximation and circle-center affine transformation sorting algorithms.

In the fringe projection profilometer system, phase extraction is divided into wrapped phase extraction and phase unwrapping. The wrapped phase extraction algorithm is mainly divided into phase shift profiling [17] and Fourier transform profiling [18,19]. Among them, phase shift profilometry completely eliminates the interference of ambient light and surface reflectivity, providing the highest measurement resolution and accuracy. Phase unwrapping algorithms are divided into two categories: spatial domain phase unwrapping algorithms [18] and time domain phase unwrapping algorithms [17,20]. The spatial phase expansion algorithm has a phase mutation phenomenon, which leads to error accumulation and is not completely reliable. Commonly used time domain phase expansion algorithms include the Gray code method [21] and the multi-frequency heterodyne method [17]. Multi-frequency heterodyne methods can perform phase unwrapping with the help of one or more additional wrapped phase images of different frequencies. The multi-frequency heterodyne method is superior to the Gray code method in terms of pattern efficiency, accuracy, and phase expansion range. Among them, Servin et al. [22] obtained two parcel phase diagrams with similar wavelengths and synthesized two phase diagrams using phase difference and phase sum at the same time. Based on Servin’s work, G.L. Du et al. proposed a three frequency or more frequency phase shifting algorithm [23] to adapt to harsh noise conditions and improve measurement accuracy or phase sensitivity. In summary, in order to enhance the anti-interference ability of the aeroengine blade measurement system, this study uses the three-frequency twelve-step phase shift method to build a fringe projection profiler.

The main research purpose of this study is to propose a new concentric ring calibration method and build a fringe projection profiler based on the three-frequency twelve-step phase shift method to make up for the shortcomings of existing aeroengine blade measurement methods.

The remainder of this article is organized as follows. Section 2 introduces the principle of multi-layer concentric ring calibration. The calibration process includes ellipse detection, circle-center approximation, and circle-center affine transformation sorting. Section 3 introduces the principle of 3D reconstruction. The experiments and results are shown in Section 4. Section 5 gives conclusions.

## 2. The Principle of Multi-Layer Concentric Ring Calibration

**Figure 1:**
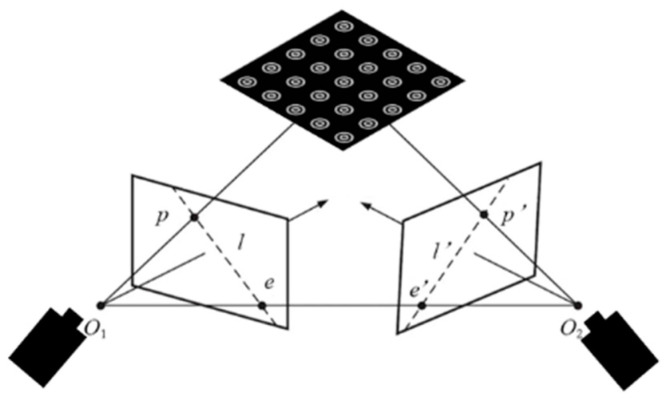
Schematic of the multi-layer concentric ring calibration.

In this section, a multi-layer concentric ring calibration method is proposed, calibration principle as shown in Figure 1. The whole process includes (1) ellipse detection, (2) circle-center approximation, and (3) circle-center affine transformation sorting.

The ellipse detection algorithm obtains the pixel coordinate-system coordinates of all ellipse centers on the multi-layer concentric ring calibration plate by introducing arc-support line segments. The circle-center approximation algorithm approximates the true circle-center position through K-means clustering, reducing the impact of the projection centrifugal error. The circle-center affine transformation calculates the coordinates of the circle center in the world coordinate system by numbering and sorting all the circle centers on the calibration plate.

### 2.1. Ellipse Detection

In image processing, an ellipse can be viewed as a superposition of several small straight line segments. These small straight line segments can be obtained by calculating the level line of each pixel through the LSD straight line detection algorithm [24]. Arc-support segments are built on these tiny straight segments. By connecting together small straight segments that meet a certain angle range, the arc formed is called an arc-support segment. The specific extraction process can be found in the literature [25].

Based on the introduction of arc support line segments, the specific process of ellipse detection includes (1) image edge detection, (2) formation of arc-support line segment groups, (3) synthesis of initial ellipse set, and (4) ellipse feature fitting.

The image edge detection algorithm can reduce the interference of the image background on ellipse extraction and enhance the edge feature information. The formation of arc-supported line segment groups can filter out the interference of non-arc line segments and speed up detection. The composition of the initial ellipse set is used to connect the divided arc-support line segment groups in the same ellipse. Ellipse feature fitting obtains the characteristic parameters of the ellipse through the nonlinear direct least squares method.

#### 2.1.1. Image Edge Detection

**Figure 2:**
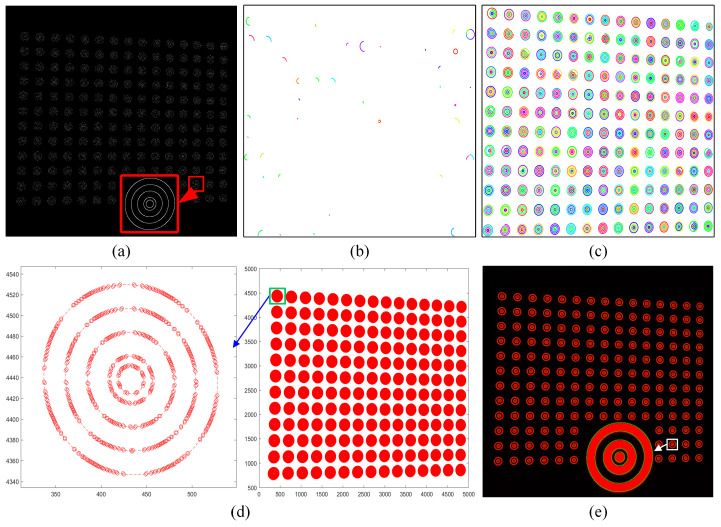
Schematic of the ellipse detection. (**a**) Concentric ellipse edge extraction based on the Canny operator. (**b**) Incomplete arc-supports segment groups. (**c**) Highly complete arc-supports segment groups. (**d**) The initial ellipse set and the enlarged point set of a certain concentric ring. (**e**) The ellipse detection final result.

This study uses a self-designed five-layer concentric ring calibration plate for calibration and uses the Canny operator [26] to perform an edge extraction algorithm on the original grayscale image. Finally, the edge binary images of 900 concentric rings are obtained, and the calculation results are shown in Figure 2a.

#### 2.1.2. Formation of Arc-Support Line Segment Groups

For any two arc-support line segments to be connected into a group, three conditions need to be met: continuity, convexity, and angular deviation range.

Continuity means that the distance between the head of one arc-support segment and the tail of another arc-support segment should be close enough. Convexity means that two connected arc-support line segments should have the same convex direction. The angle deviation range means that the angle deviation between two consecutive arc-support line segments should be less than the specified range.

On the basis of meeting the conditions, it is also necessary to set evaluation indicators for the formed arc-support line segment group. The span angles of different arc-supported line segment groups are different. The span angles of some arc-supported line segment groups are very small, and some arc-supported line segment groups have approximately formed an ellipse, as shown in Figure 2b,c.

The formula for calculating the span angle of the arc supporting line segment group is:

(1) $$\theta^{i}=\sum_{j=1}^{n-1}\theta_{j}^{i}$$

(2) $$S^{i}=(\sum_{j=1}^{n-1}\theta_{j}^{i})/360{}^{\circ}$$

where *i* represents the i-th arc supporting the line segment group, *j* represents the j-th arc supporting the line segments in the group, and $S^{i}$ represents the normalized angle value.

#### 2.1.3. Synthesis of Initial Ellipse Set

The arc-support line segment group may have the following two situations. (1) An arc-support group may contain all arc-support line segments of an ellipse. (2) Although one arc-support line segment group and another arc-support line segment group belong to the same ellipse, they are far apart.

Therefore, we synthesize the initial ellipse set from both local and global aspects. In the local aspect, the arc-support line segment group with a large span angle is used as an initial ellipse set. For example, some arc-support line segment groups with a span angle close to 360 degrees are used. In the global aspect, it is to pair the arc-support line segment groups that meet the conditions. The conditions that need to be met are (1) polarity constraints, (2) area restrictions, and (3) adaptive inliers criterion.

Polarity constraints mean that only arc-support line segment groups with the same polarity are paired. The reason is that the inside of an ellipse is always lighter or darker than the outside, where lighter means positive polarity and darker means negative. Therefore, if multiple arc-support segment groups belong to the same ellipse, their polarities must be the same.

Area restriction refers to calculating the matching range of the line segment groups supported by each arc. Only groups within the matching range can be paired.

The adaptive inliers criterion means that the number of pixels of an ellipse fitted by multiple arc-support line segment groups passing through multiple groups is greater than the length of any arc-support line segment group. The formula is:

(3) $$\#\{p_{i}:p_{i}\in \mathrm{SI}(L_{j})\}>\mathrm{Length}(L_{j})$$

In summary, the initial ellipse set is synthesized for the arc-support line segment group, and the calculation results are as shown in Figure 2d.

#### 2.1.4. Ellipse Feature Fitting

Nonlinear least squares (NLS) is a method used to fit nonlinear models. The goal of this method is to minimize the squared difference between the actual observed values and the fitted values. This study uses Levenberg–Marquardt for the numerical iterative solution of nonlinear fitting, and the parameters are adjusted at each iteration to reduce the squared error. The Levenberg–Marquardt algorithm uses a parameter λ, called the damping factor, to balance the steepest descent method and Newton’s method. When λ is small, the algorithm is similar to Newton’s method. When λ is large, the algorithm is similar to the steepest descent method.

This algorithm combines the advantages of the steepest descent method and Newton’s method and can converge to the optimal solution faster. All concentric ellipses are fitted by the nonlinear direct least squares method, and data that do not fit the ellipse or ellipses whose long axis is too large are deleted in reverse order. The fitted ellipse is shown in Figure 2e.

### 2.2. Circle-Center Approximation

**Figure 3:**
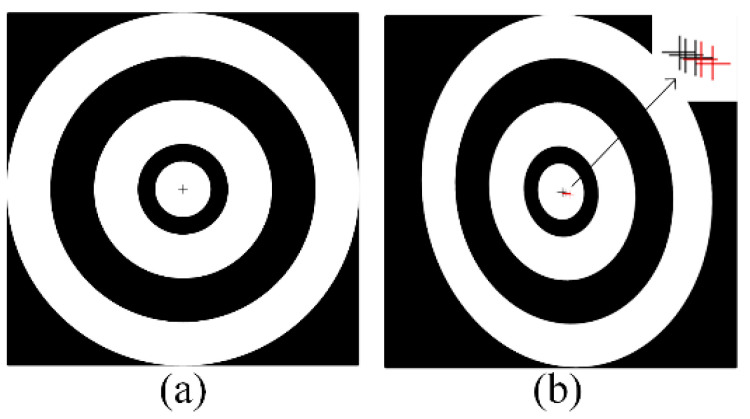
Schematic of the projection centrifugal error. (**a**) Five concentric rings have the same center. (**b**) After perspective projection, the center of the circle is shifted. Different colors represent different center positions of the five concentric rings.

As shown in Figure 3, the center of the projected ellipse does not coincide with the center of the actual projected circle due to the perspective projection transformation of the space circle. This deviation is called the projection centrifugal error [27].

In order to eliminate the impact of the projection centrifugal error on obtaining the true circle-center coordinates as much as possible, we use the K-means clustering algorithm to approximate the true circle-center coordinates.

The goal of the K-means is to minimize the square distance between the internal data of each cluster and the average value of the internal data of the cluster and to make the distance measured between different clusters as large as possible [28].

The algorithm first selects K points as the starting cluster centers. Then, by calculating the distance between all sample points and each cluster center, each sample point is assigned to the cluster center closest to it, thereby forming K clusters. Next, the clustering center is moved, and the average of the K clusters generated in the previous step is calculated as the new clustering center.

The Euclidean distance-calculation formula between all sample points in a certain cluster and the cluster center point is:

(4) $$d\left(X,C\right)=\sqrt{\sum_{i=1}^{m}{\left(X_{i}-C\right)}^{2}}$$

where *X* is all the sample points in a certain cluster; *C* is the cluster center point of the cluster; and *m* is the number of all sample points in the cluster.

Then, continue to perform the cluster assignment, move the cluster center, and iterate multiple times until convergence (the center point no longer changes, or the specified number of iterations is reached). Then, the clustering process ends.

Lastly, assess the excellence of the clustering algorithm outcomes. This can be achieved by computing the sum of squared errors (SSE) for the concentric ring center point set. It is calculated by summing the squares of the Euclidean distances between each data point and its corresponding cluster center. Its calculation formula is:

(5) $$SSE=\sum_{i=1}^{k}\sum_{X\in C_{i}}{\left|d\left(X,C_{i}\right)\right|}^{2}$$

The *SSE* quantifies the dispersion among data points and their respective cluster centers. A lower *SSE* value indicates superior clustering, implying that the data points are closely grouped around their respective centers.

### 2.3. Circle-Center Affine Transformation Sorting

**Figure 4:**
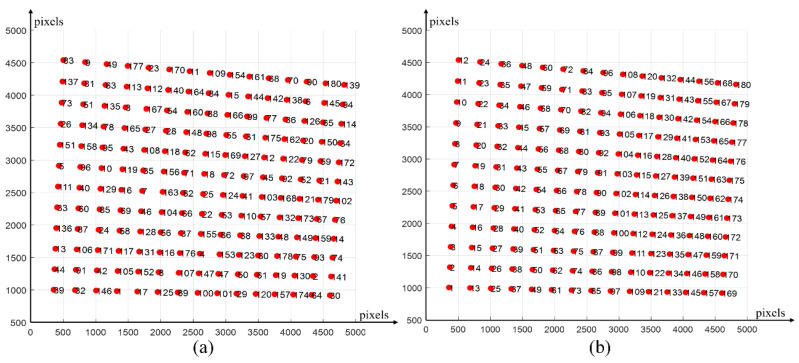
Schematic of the circle-center affine transformation sorting. (**a**) Unordered point set. (**b**) Ordered point set.

During the calibration process of the binocular camera, it is necessary to number and sort all the circle centers on the calibration board to obtain the world coordinate system. In order to solve this problem, we propose a center–center affine transformation sorting algorithm for camera calibration. In Figure 4a, the center coordinates of the concentric circles with randomly distributed serial numbers are shown.

The algorithm first finds the diagonal corner points of the point set according to the order of the horizontal and vertical coordinates of the quadrilateral point set and determines whether all other points are on the same side of the diagonal straight line. If it is, it is the corner point on the same side. Then, go through the corner point to find its two adjacent edges. Calculating the coordinates of the intersection point based on the edge line of one corner point and the edge line of the opposite corner point is the other corner point.

After the order of the four corner points is determined, the perspective transformation matrix is calculated through the predetermined corresponding four-point coordinates, and then, all points are mapped to the square through perspective transformation.

Finally, the point set on the quadrilateral is sorted according to the principle of “bottom to top, left to right”, and the sorted index is applied to the original unordered point set to complete. Apply the 180 sorted indexes to the original unordered point-set coordinates and draw them one by one in order. The sorting results are shown in Figure 4b.

## 3. Principles of 3D Reconstruction

**Figure 5:**
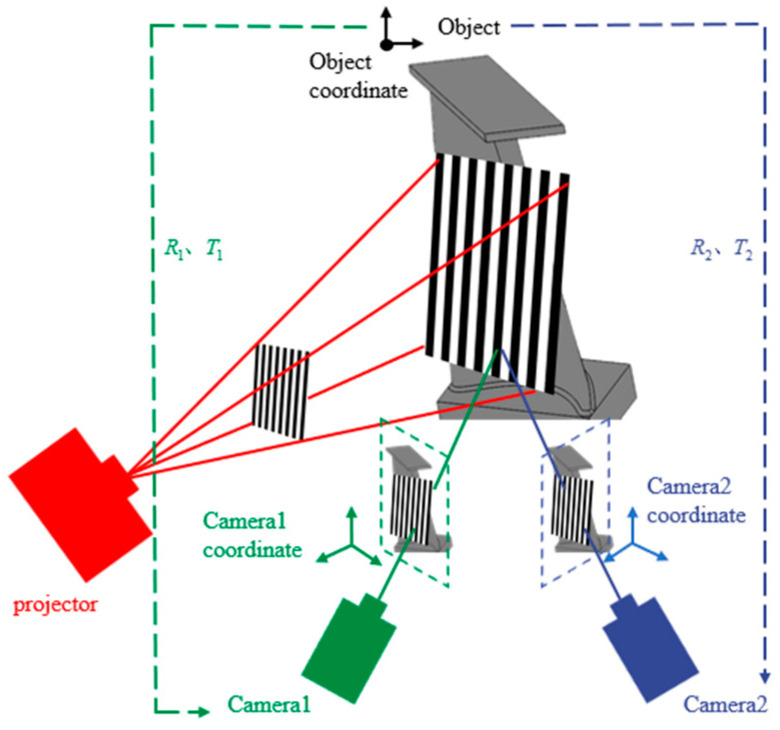
The fringe projection profilometer. Fringe patterns are projected to the blade by the projector. Binocular cameras capture modulated stripe patterns.

For the measurement of aeroengine blades, this section introduces a three-dimensional reconstruction scheme measurement method based on the three-frequency twelve-step phase shift method, as shown in Figure 5. The specific process of this method includes (1) wrapped phase extraction, (2) phase unwrapped, and (3) sub-pixel stereo matching.

The wrapping phase extraction based on the twelve-step phase shift method and the phase unwrapping based on the multi-frequency heterodyne method is both to inversely solve the phase value of each pixel in the image projection area. Sub-pixel stereo matching based on cubic spline interpolation performs pixel-level matching on the projection area based on phase values and, then, calculates the depth through the triangulation principle.

### 3.1. Wrapped Phase Extraction

In order to solve the phase value φ(x,y), the phase shift value $△\varphi_{k}$ needs to be introduced to establish a system of equations. The formula is:

(6) $$I(x,y)=A+B\cos\left(\varphi (x,y)+△\varphi_{k}\right)$$

where *A* is the average grayscale of the image, *B* is the grayscale modulation degree of the image, and $△\varphi_{k}$ are the relative phase code value and phase shift value respectively.

$△\varphi_{k}$ is a known quantity, *A*, *B* and φ(x,y) are three unknown parameters. Therefore, at least three equation solutions need to be calculated to obtain the unique solution. In this section, the number of phase shift steps N is introduced to be 12, so it is called the twelve-step phase shift method.

According to the trigonometric formula, the light-intensity formula of the phase-shifted fringe image can be simplified:

(7) $$\begin{array}{l} I_{k}(x,y)=A(x,y)+B(x,y)\cos\left[\varphi (x,y)+\frac{2k\pi }{N}\right]\\ =A(x,y)+B(x,y)\times \left[\cos(\varphi (x,y))\cos\left(\frac{2k\pi }{N}\right)-\sin(\varphi (x,y))\sin\left(\frac{2k\pi }{N}\right)\right]\\ =A(x,y)+\cos\left(\frac{2k\pi }{N}\right)\cdot \underset{B_{1}(x,y)}{\underbrace{B(x,y)\cos(\varphi (x,y))}}-\sin\left(\frac{2k\pi }{N}\right)\cdot \underset{B_{2}(x,y)}{\underbrace{B(x,y)\sin(\varphi (x,y))}} \end{array}$$

The final wrapping phase calculation formula of the phase shift method is as follows:

(8) $$\varphi (x)=\arctan\left(\frac{B_{2}(x,y)}{B_{1}(x,y)}\right)=-\arctan\left[\frac{\sum_{k=0}^{N-1}\left(I_{k}\sin(2k\pi /N)\right)}{\sum_{k=0}^{N-1}\left(I_{k}\cos(2k\pi /N)\right)}\right]$$

According to the above calculation formula, it can be concluded that the phase is an arctangent function, which is periodic, and its period is 2π. So, it is called the wrapped phase.

### 3.2. Phase Unwrapped

Since the neighbor phase is periodic, the corresponding neighbor phase value is not unique for all pixels in the same row. Therefore, the multi-frequency heterodyne method is introduced to turn the neighbor phase into a continuous absolute phase.

The basic idea of the multi-frequency heterodyne method is to heterodyne two sinusoidal signals with a small frequency difference to obtain an alternating signal with a lower frequency, thereby achieving phase unwrapping of the original signal. The specific calculation formula is:

(9) $$\varphi_{12}=\varphi_{1}-\varphi_{2}\Rightarrow \frac{1}{T_{12}}=\frac{1}{T_{1}}-\frac{1}{T_{2}}\Rightarrow T_{12}=\frac{T_{1}T_{2}}{T_{2}-T_{1}}$$

where *φ*_1_ and *φ*_2_ are the phase functions of two small periods. *φ*_12_ is the phase function with a larger period calculated by the heterodyne method of *φ*_1_ and *φ*_2_, and its value is the least common multiple of *φ*_1_ and *φ*_2_.

Similarly, the computation of the multi-frequency heterodyne can be derived from the outcome of the dual-frequency heterodyne, thereby attaining the capability to unfold the global phase effect.

(10) $$T_{23}=\frac{T_{2}T_{3}}{T_{3}-T_{2}}$$

(11) $$T_{123}=\frac{T_{12}T_{23}}{T_{23}-T_{12}}$$

To minimize the computational load of binocular stereo matching, it is imperative to compute the modulation degree of the entire image. The modulation degree quantifies the magnitude of change in the grayscale value of the projected stripe pattern and enables differentiation between the background and projection areas. The calculation formula for modulation degree is expressed as follows:

(12) $$B(x,y)=\frac{2}{N}\times \sqrt{{\left[\sum_{k=0}^{N-1}\left(I_{k}(x,y)\sin(2k\pi /N)\right)\right]}^{2}+{\left[\sum_{k=0}^{N-1}\left(I_{k}(x,y)\cos(2k\pi /N)\right)\right]}^{2}}$$

### 3.3. Sub-Pixel Stereo Matching

Since the projector and the projected object cannot be completely perpendicular, the phase values of the left camera and the right camera at the same feature point will deviate. In order to solve this problem, this section introduces a sub-pixel phase value matching algorithm based on cubic spline interpolation.

**Figure 6:**
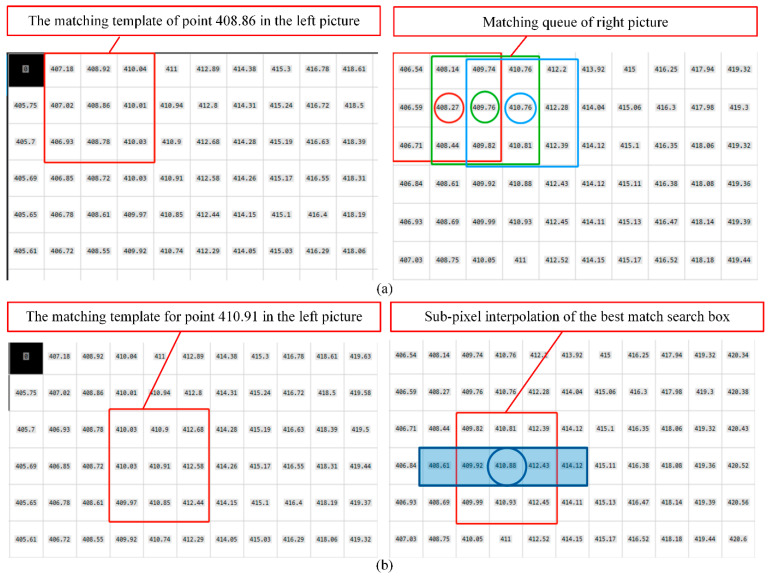
Schematic of the sub-pixel stereo matching (**a**) Select any point on the left phase diagram to create a 3 × 3 matching template, and search for a matching queue on the right phase diagram. (**b**) Select the two pixels before and after the best matching search box to perform cubic spline interpolation.

First, epipolar correction [29] is introduced to correspond to the feature points of the left and right cameras on the same line. Then, randomly select a point in the left phase image to create a 3 × 3 matching template. Create a 3 × 3 search box on the same row of the right phase map and calculate the similarity, as shown in Figure 6a.

This section uses the absolute deviation between the template and the image to calculate the similarity:

(13) $$\mathrm{sad}(x,y)=\frac{1}{9}\sum_{(u,v)\in T}|L(u,v)-R(u+x,v+y)|$$

The search boxes whose similarities are within the threshold range form a matching queue. Among them, the one with the greatest similarity in the matching queue is the best-matching search box.

Finally, the two pixels before and after the best-matching search box are selected to perform cubic spline interpolation, and the sub-pixel coordinates of the center pixel value of the left search box in the row on the right are obtained, as shown in Figure 6b. Cubic spline interpolation divides the function within the interpolation interval into segments, and each segment is described by a cubic polynomial [30].

## 4. Experiment

**Figure 7:**
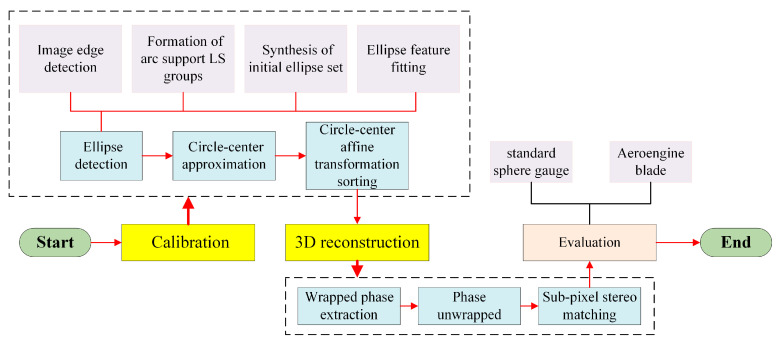
The flow chart of the entire measurement process.

The measurement process starts with camera calibration based on multi-layered concentric rings, then completes three-dimensional reconstruction through wrapped phase extraction, phase unwrapping, and sub-pixel stereo matching. Finally, the standard sphere gauge and blade are evaluated, respectively. The flow chart of the entire measurement process is shown in Figure 7.

The equipment used in the experiment includes an engine blade; a standard spherical gauge; two calibration plates, including a multi-layer concentric ring calibration plate and a checkerboard calibration plate; two industrial cameras; a projector; and a precision air flotation turntable. The resolution of the two industrial cameras is 5120 (H) and 5120 (V). The frame rate is 15.1 FPS, and the pixel size is 2.5 μm × 2.5 μm. The projector resolution is 1280 × 720.

Based on the above experimental equipment, a total of two experiments were designed. Experiment 1 uses a multi-layer concentric ring calibration plate and a checkerboard calibration plate to calibrate the camera and uses a standard spherical gauge to verify the measurement accuracy of the two calibration results. Experiment 2 uses the fringe projection profilometer to scan the aeroengine blade based on the calibration method proposed in this article and compares it with the three-dimensional coordinate measuring machine to evaluate the measurement accuracy of the blade chord length.

### 4.1. Calibration Comparison Experiment

The center distance of the standard ball selected for the experiment is 99.925 mm. The uncertainty of the measurement results is 0.002 mm, and the inclusion factor is 2.

**Figure 8:**
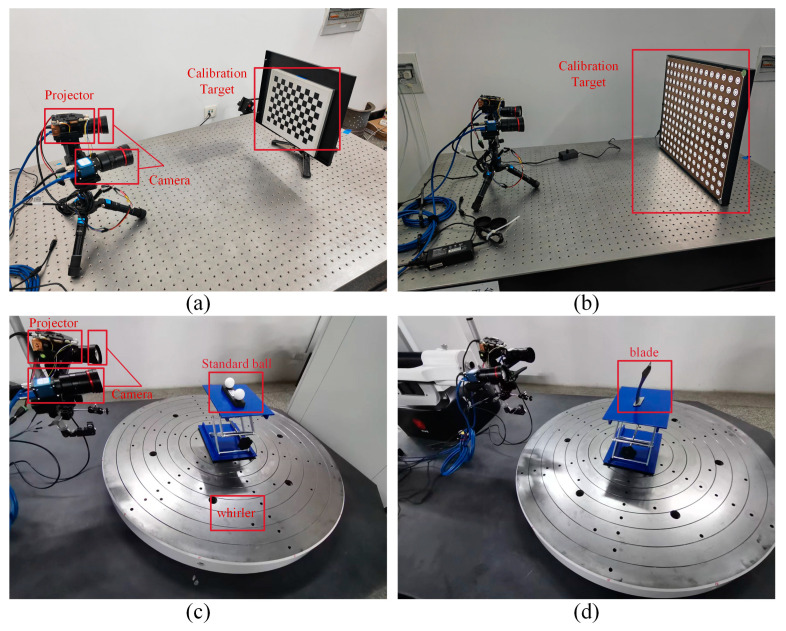
Measuring equipment for experiments. (**a**) Checkerboard calibration experiment. (**b**) Multi-layer concentric ring calibration experiment. (**c**) Standard spherical gauge measurement experiment for comparing calibration accuracy. (**d**) Blade measurement experiment.

The experiment measures the three-dimensional reconstruction accuracy of the system by measuring the center-distance error of the standard ball at any 10 positions in the measurement space. First, the camera is calibrated using multi-layer concentric ring calibration plates and checkerboard calibration plates, respectively, as shown in Figure 8a,b.

**Figure 9:**
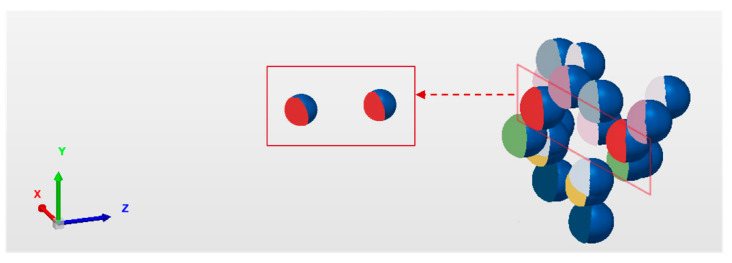
Schematic of the standard sphere point cloud. The dark-blue sphere represents the sphere fitted by each set of point clouds. Semicircles of different colors represent standard sphere point cloud data collected at different locations.

Then, the standard sphere is reconstructed three-dimensionally through a fringe projection profilometer, as shown in Figure 8c. Finally, the center position of the standard sphere is fitted by the least squares method, and the distance between the centers of the spheres is calculated using the Euclidean geometric distance formula, as shown in Figure 9.

It is known that the center distance of a standard ball is 99.925 mm. Table 1 presents the absolute and average deviations of the distance between the centers of the ball at ten different positions, as obtained by the two calibration methods. In the table, the absolute deviation is abbreviated as AD, and the mean absolute deviation is abbreviated as MAD.

It can be seen from the calculation data in the table that the three-dimensional reconstruction error of the standard ball under multi-layer concentric ring calibration is 0.02352 mm, and the three-dimensional reconstruction error of the standard ball under checkerboard calibration is 0.08327 mm. After multi-layer concentric ring calibration, the measurement accuracy of the three-dimensional reconstruction is increased by 3–4 times, meeting the accuracy requirements of the three-dimensional reconstruction of aeroengine blades.

### 4.2. Aeroengine Blade Measurement Experiment

Based on the multi-layer concentric ring calibration results, a multi-view three-dimensional reconstruction of the aeroengine blade is performed. In the experiment, the chord length value of the blade under a certain height section was selected as the parameter to be measured, and the measurement accuracy of the fringe projection profiler was measured by comparing the measurement values of the fringe projection profiler and the three-dimensional coordinate measuring machine.

**Figure 10:**
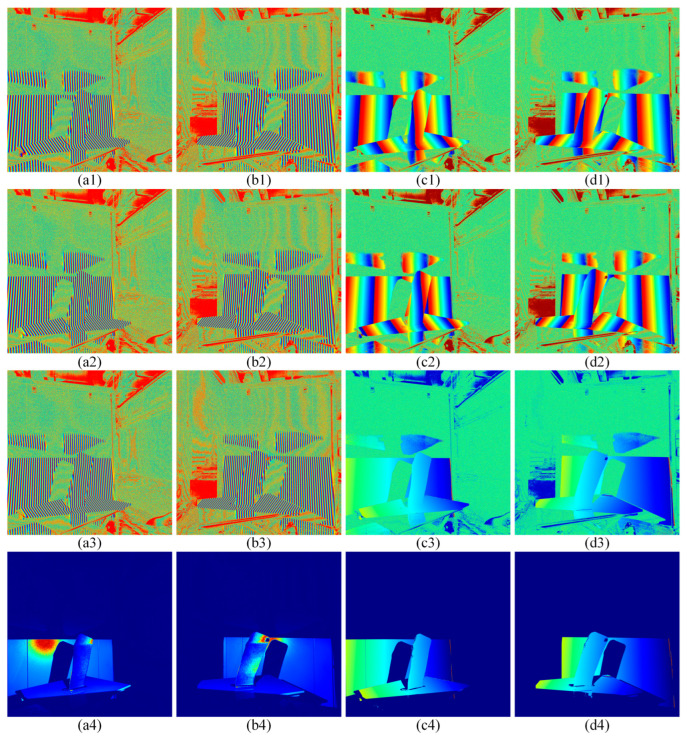
Different colors within the projection area represent different phase values. (**a1**–**a3**,**b1**–**b3**) Left and right wrapped phase diagrams at three frequencies. (**a4**,**b4**) Left and right modulation diagram. (**c1**,**c2**,**d1**,**d2**) Left and right first unwrapped phase diagram. (**c3**,**d3**) Left and right second unwrapping phase diagram. (**c4**,**d4**) Left and right phase distribution diagram after modulation degree filtering.

Initially, the twelve-step phase shift method is employed to compute wrapped phases with periods of T1 = 28, T2 = 26, and T3 = 24, measured in pixels of the projection light machine. The resulting left and right wrapped phase diagrams at three frequencies are illustrated in Figure 10a1–a3,b1–b3.

Then, calculate the modulation degree of the left camera and the right camera to form a mask area, as shown in Figure 10a4,b4.

Then, the multi-frequency heterodyne method is used to perform the first unwrapping of T1 = 28, T2 = 26, and T3 = 24, respectively. The results are recorded as φ12 and φ23, as shown in Figure 10c1,c2,d1,d2. Then, the second unwrapped phase is performed on φ12 and φ23, and the result is recorded as φ123, as shown in Figure 10c3,d3.

Finally, modulation filtering is performed on the second unwrapped phase image Figure 10c3,d3 using the modulation mask images in Figure 10a4,b4, and the results are shown in Figure 10c4,d4. Stereo matching is performed based on the phase distribution map to achieve a three-dimensional reconstruction of the blade under a single viewing angle.

**Figure 11:**
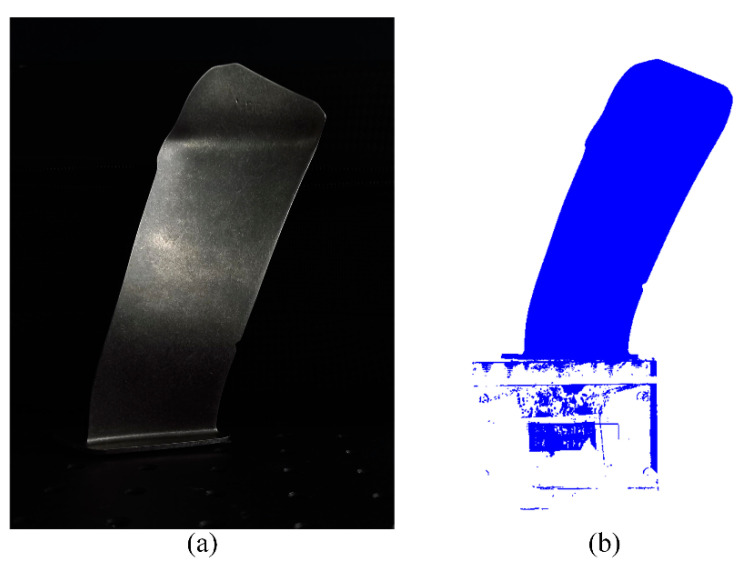
(**a**) Original image of the blade. (**b**) 3D reconstruction of the blade.

Due to the limited scanning range of structured light, it is necessary to use a precision rotating table to drive the workpiece from multiple angles to achieve complete measurement. Therefore, multi-angle point clouds are spliced and unified into the same coordinate system through the ICP algorithm [31]. Finally, the original point cloud of the multi-view scan of the aeroengine blade is obtained, as shown in Figure 11.

**Figure 12:**
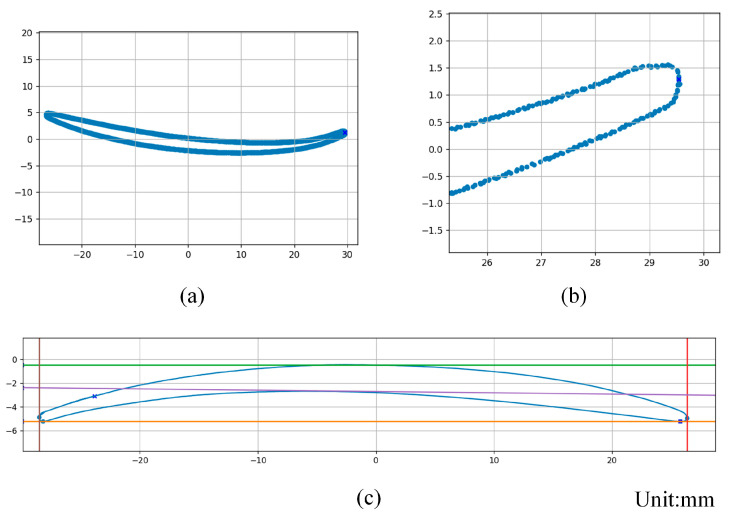
(**a**) Cross-section point cloud at a certain height. (**b**) Enlarged view of the end of the section point cloud. (**c**) Blade cross-section chord search results.

Based on the multi-view scanning of the original point cloud of the blade, the PCA algorithm [32] was used to benchmark the original point cloud of the aeroengine fan blade and establish a feature coordinate system. Then, cross-sectional projections at different heights are taken from the original point cloud and processed by PCA. Figure 12a,b shows the cross-sectional point cloud at a certain height.

As shown in Figure 12c, the aforementioned iterative search method is used to calculate the chord length from the blade cross-section point cloud. The measurement results are shown in Table 2. In the table, the absolute deviation is abbreviated as AD, and the mean absolute deviation is abbreviated as MAD.

The average deviation of the chord length is 0.064 mm, and the maximum absolute deviation is 0.092 mm. During the entire measurement process, 3 × 12 pictures need to be taken from a single viewing angle, where 3 is the frequency number of the multi-frequency heterodyne method, and 12 is the number of steps of the phase shift method. Through CUDA parallel acceleration, the acquisition and reconstruction time at a single perspective can be controlled within 0.4 s, and the multi-view measurement with the turntable takes about 10 min in total.

The blade used in this study is a compressor blade, and Table 3 lists the contour tolerance of the blade’s mid-section. The chord length tolerance under different accuracy requirements is determined according to the Formula (14).

(14) $$\delta_{b}=4\delta_{y}$$

where $\delta_{y}$ is the contour tolerance and *δ_b_* is the chord length tolerance.

Commonly used rotor blades generally use level 2–3 accuracy, and stator blades generally use level 3–4 accuracy. The chord length tolerance corresponding to level 2 accuracy is 0.80 mm, so this three-dimensional reconstruction solution meets the measurement accuracy requirements of aeroengine blades.

## 5. Conclusions

In this study, we aim to improve the accuracy of existing aeroengine blade measurement methods while achieving comprehensive measurements. Our findings can be summarized as follows:

(1) This study proposes a new concentric ring calibration method and designs a multi-layer concentric ring calibration plate. This method first obtains the centers of all ellipses on the calibration plate based on the arc-supported line-segment segmentation algorithm and, then, eliminates perspective projection errors through the K-means center approximation algorithm proposed in this article. Finally, the sequence of the circle centers on the calibration plate is obtained through the circle-center affine transformation sorting proposed in this article. Experiments have proven that, compared with the checkerboard calibration method, the measurement accuracy of the multi-layer concentric ring calibration method can be improved by 3–4 times;
(2) Based on the above calibration method, this study built a fringe projection profilometer based on the three-frequency twelve-step phase shift method to conduct all-round measurements of aeroengine blades. Through comparative experiments between the design and the coordinate measuring machine (CMM), the average deviation of the blade chord length was measured to be 0.064, and the maximum absolute deviation was 0.092. This three-dimensional reconstruction scheme meets the measurement index requirements of aeroengine fan blades.

Due to the high reflectivity of the surface of aeroengine blades, light-intensity saturation is prone to occur, which leads to the distortion of phase information in the saturated area and seriously affects the accuracy of three-dimensional measurement. Therefore, we hope that, in subsequent research work, we can design a specific image-processing algorithm to repair the light-intensity saturation phase error.

## Figures and Tables

**Table 1:** Calibration comparison experiment results.

NO.	Concentric Ring Calibration		Checkerboard Calibration	
	Ball Center Distance (mm)	AD	Ball Center Distance (mm)	AD
1	99.9016	0.0234	100.0435	0.1185
2	99.9552	0.0302	100.0347	0.1097
3	99.8915	0.0335	99.9944	0.0694
4	99.9095	0.0155	99.8585	0.0665
5	99.9460	0.0210	100.0032	0.0782
6	99.9379	0.0129	99.9794	0.0544
7	99.9548	0.0298	100.0215	0.0965
8	99.9315	0.0065	100.0249	0.0999
9	99.8950	0.0300	100.0183	0.0933
10	99.9574	0.0324	99.9713	0.0463
MAD	0.02352		0.08327	

**Table 2:** Blade chord measurement results.

NO.	Reference (mm)	Measurements (mm)	AD
1	54.9048	54.8408	0.064
2	55.1291	55.1821	0.053
3	55.6643	55.5813	0.083
4	55.9587	55.9867	0.028
5	56.4997	56.4077	0.092

**Table 3:** The contour tolerance of the blade mid-section.

Accuracy Level	Contour Tolerance of the Blade Mid-Section mm
1	0.16
2	0.20
3	0.25
4	0.31

## Data Availability

All data can be provided upon reasonable request to the corresponding author.
